# Enhancing cytokinin synthesis by overexpressing *ipt* alleviated drought inhibition of root growth through activating ROS-scavenging systems in *Agrostis stolonifera*

**DOI:** 10.1093/jxb/erw019

**Published:** 2016-02-17

**Authors:** Yi Xu, Patrick Burgess, Xunzhong Zhang, Bingru Huang

**Affiliations:** ^1^Department of Plant Biology and Pathology, Rutgers University, New Brunswick, NJ 08901, USA; ^2^Department of Crop and Soil Environmental Sciences, Virginia Polytechnic Institute and State University, Blacksburg, VA 24061, USA

**Keywords:** Antioxidant, drought stress, qRT-PCR, root respiration, ROS scavenging system, turfgrass.

## Abstract

Drought stress limits root growth and inhibits cytokinin (CK) production. Increases in CK production through overexpression of isopentenyltransferase (*ipt*) alleviate drought damages to promote root growth. The objective of this study was to investigate whether CK-regulated root growth was involved in the alteration of reactive oxygen species (ROS) production and ROS scavenging capacity under drought stress. Wild-type (WT) creeping bentgrass (*Agrostis stolonifera* L. ‘Penncross’) and a transgenic line (S41) overexpressing i*pt* ligated to a senescence-activated promoter (*SAG12*) were exposed to drought stress for 21 d in growth chambers. *SAG12-ipt* transgenic S41 developed a more extensive root system under drought stress compared to the WT. Root physiological analysis (electrolyte leakage and lipid peroxidation) showed that S41 roots exhibited less cellular damage compared to the WT under drought stress. Roots of *SAG12-ipt* transgenic S41 had significantly higher endogenous CK content than the WT roots under drought stress. ROS (hydrogen peroxide and superoxide) content was significantly lower and content of total and free ascorbate was significantly higher in S41 roots compared to the WT roots under drought stress. Enzymatic assays and transcript abundance analysis showed that superoxide dismutase, catalase, peroxidase, and dehydroascorbate reductase were significantly higher in S41 roots compared to the WT roots under drought stress. S41 roots also maintained significantly higher alternative respiration rates compared to the WT under drought stress. The improved root growth of transgenic creeping bentgrass may be facilitated by CK-enhanced ROS scavenging through antioxidant accumulation and activation of antioxidant enzymes, as well as higher alternative respiration rates when soil water is limited.

## Highlight

The relationship between an *adenine isopentenyltransferase* transgene, reactive oxygen species (ROS) content and the ROS-scavenging system, mechanistically contributing to improved root growth under drought stress in creeping bentgrass, is discussed.

## Introduction

Drought stress is a primary limiting factor of plant growth and productivity in semi-arid and arid climatic areas. Drought inhibition of shoot and root growth has been associated with the induction of oxidative damage due to over-production of reactive oxygen species (ROS), such as superoxide (O_2_^-^) and hydrogen peroxide (H_2_O_2_) (Halliwell, 2006). Excessive ROS can lead to lipid peroxidation, protein degradation, and nucleotide damage further inhibiting a wide range of plant cellular processes (Smirnoff, 1993; Kültz, 2005, Halliwell, 2006; Anjum *et al.*, 2011). Therefore, suppressing ROS production or enhancing the capacity for ROS scavenging by antioxidant enzymes [i.e. superoxide dismutase (SOD), catalase (CAT), peroxidase (POD), ascorbate peroxidase (APX), monohydroascorbate reductase (MR), dehydroascorbate reductase (DR), and glutathione reductase (GR)] and antioxidant compounds [i.e. ascorbate (ASA) and glutathione (GSH)] can suppress drought-induced oxidative damage (Asada 1992; Bowler *et al.*, 1992; Willekens *et al.*, 1997; Noctor and Foyer, 1998; Mittler 2002). Despite extensive knowledge of ROS scavenging regulating shoot responses to drought stress, limited information is available describing mechanisms of ROS detoxification for maintaining root growth during drought stress. ROS are mainly produced in root mitochondria during respiration; the cytochrome respiration pathway involves electron transfer from the ubisemiquinone radical to oxygen, prompting superoxide formation (Halliwell and Gutteridge, 1999). Activating both enzymatic and non-enzymatic antioxidant defense systems could suppress drought-induced ROS formation in roots. In addition, alternative respiration pathways, which divert the high-energy electrons to alternative oxidase can also avoid ROS over-production in roots (Purvis and Shewfelt, 1993; Wagner and Krab, 1995). However, it is uncertain how to maintain active ROS defense systems in roots that are typically weakened by prolonged drought stress (Jain *et al.*, 2006).

Cytokinins (CK) are a class of plant hormones synthesized primarily in actively growing root tips, which play critical roles in regulating plant growth and responses to abiotic stresses (Li *et al.*, 1992; Peres and Kerbauy, 1999; Takei *et al.*, 2001). Drought stress inhibits CK synthesis and accelerates CK degradation, reducing CK levels in roots and shoots (Kudoyarova *et al.*, 2007; Nishiyama *et al.*, 2011). Increasing endogenous CK content through exogenous application of CK (Zhang and Ervin, 2004; Zavaleta-Mancera *et al.*, 2007; Baque *et al.*, 2010) or genetic modification to overexpress isopentenyltransferase (*ipt*) controlling cytokinin synthesis has positive effects on improving plant drought tolerance, which has been attributed to the promotion of photosynthesis, water use efficiency, and antioxidant metabolism of shoots in various plant species (Zhang and Ervin, 2004; Rivero *et al.*, 2007, 2009; Merewitz *et al.*, 2010, 2011a; Ghanem *et al.*, 2011). CK have been shown to modulate leaf enzymatic antioxidant activities (i.e. POD, SOD, and CAT), activating leaf defenses to abiotic stresses (Chaloupková and Smart 1994; Kurepa *et al.* 1997; Petit-Paly *et al.* 1999; Synkova *et al.*, 2006; Zavaleta-Mancera *et al.*, 2007). In addition, Zhang and Ervin (2008) also reported antioxidant properties of CK, protecting leaves from stress-induced oxidation. Most of previous work including the aforementioned literature on CK regulation of drought tolerance has focused on enhancing shoot growth and suppressing leaf senescence.

Few studies have examined CK-regulation of root responses to drought stress involving oxidative stress. CK have been widely reported to play negative roles in primary root elongation and lateral root formation of dicot species, such as Arabidopsis, under non-stress conditions (Werner *et al.*, 2003; Kuderová *et al.*, 2008). However, increasing CK content promoted growth of fibrous root systems in monocot species, such as barley (*Hordeum vulgare*) through RNAi silencing of *HvCKX1* encoding cytokinin oxidase catalyzing CK cleavage (Zalewski *et al.*, 2010) and creeping bentgrass through overexpressing *ipt* gene under drought stress (Merewitz et al., 2010, 2011a, *b*). The *PSARK::IPT* transgenic tobacco plants with increased CK content also exhibited greater root biomass than the WT under drought stress (Rivero *et al.*, 2007). The mechanisms of CK-promotion of root growth under drought stress, with particular focus on whether CK mediates antioxidant defense systems protecting roots from drought-induced oxidative damages, are not well understood.

Since drought-inhibition of root growth is associated with the production of ROS, and CK may enhance root growth under drought stress, it is reasonable to postulate that CK may contribute towards maintaining or enhancing root growth under drought stress or alleviate drought damages in roots by suppressing ROS accumulation, increasing production of non-enzymatic antioxidant compounds, and/or activating enzymatic ROS scavenging systems. Therefore, the objective of this study was to investigate whether CK-regulated root growth under drought stress was involved in the alteration of reactive oxygen species (ROS) production and ROS scavenging capacity using a perennial grass species, creeping bentgrass, overexpressing *ipt* ligated to a senescence-associated promoter (*SAG12*). Previous work in our lab revealed improvement in drought tolerance and associated proteins and metabolites involved in the CK-regulation of shoot growth by increasing endogenous CK content in the *SAG12-ipt* creeping bentgrass lines (Merewitz *et al.*, 2010, 2011a, b, 2012). The present study focuses on examining root tolerance to drought stress and antioxidant metabolism of roots in *SAG12-ipt* creeping bentgrass, which may enable transgenic plants to maintain active root growth under prolonged periods of drought stress.

## Materials and methods

### Plant materials and growth conditions

The plants tested in this experiment were *A. stolonifera* cv. Penncross wild-type line WT, which was transformed with the empty vector used for *SAG12-ipt* transgene, and *SAG12-ipt* transgenic line S41. S41 was examined in this study as previous studies have shown that this transgenic line exhibited superior drought tolerance, as manifested by greater overall turf quality, improved root viability, and increased root iPA and total CK content, as well as other physiological factors compared to the WT (Merewitz *et al.*, 2010, 2011a). Four individual plants, each of which contained 30 uniform-size tillers of either WT or S41, were transferred to a plastic container (57×44×30cm; 12 drainage holes in base) filled with fritted clay medium (Profile Products, Deerfield, IL). Both WT and S41 were planted in eight containers (of which four were for well-watered conditions and four for were for drought stress conditions). Plants were established for 35 d in a greenhouse maintained at an average day/night temperature of 23/20°C and 780 µmol m^−2^ s^−1^ photosynthetically active radiation (PAR) at the canopy level from natural sunlight and supplemental lighting. Plants were irrigated daily, fertilized twice per week with half-strength Hoagland’s nutrient solution (Hoagland and Arnon, 1950), and trimmed to 2cm once per week during establishment. Plants were not trimmed during the final week of establishment to allow for sufficient foliar regrowth prior to stress imposition. After 35 d of plant establishment, plants were transferred to four controlled-environment growth chambers (Environmental Growth Chamber, Chagrin Falls, Ohio) and acclimated in the chambers for a week before drought stress was imposed. The growth chambers were set to maintain 22/18°C (day/night), 60% relative humidity, 12-h photoperiod, and 650 µmol m^−2^ s^−1^ PAR at the canopy level.

### Drought treatments and experimental design

Non-stressed control plants were continually supplied with adequate water and nutrients as previously described, while other plants were subjected to drought stress by withholding irrigation for 21 d. Soil volumetric water content was measured using the time domain reflectometry method (Trase TDR, Soil Moisture Equipment, Santa Cruz, CA) By 21 d of drought stress, soil water content declined to 7.8% while soil water content was maintained at 28.1% under well-watered conditions for both the WT and transgenic plants.

The experiment was arranged as a completely randomized design with each plant line (WT or S41) and each watering treatment (well-watered or drought stress) replicated four times (four containers per treatment). Plants were randomly placed in four growth chambers.

### iPa extraction, purification, and analysis

Isopentenyladenine (iPA) was extracted from roots and purified using the procedure as described by Zhang *et al.* (2013). Briefly, root tissues were ground with a mortar and a pestle in liquid nitrogen and a sample (50mg) was mixed with 1.8ml Na-phosphate buffer (50mM, pH 7.0) containing 0.02% sodium diethyldithiocarbamate as an antioxidant and iPA was extracted by continuous shaking for 1h at 4ºC. The pH for each sample was adjusted to ~2.6, and then the sample was slurried with Amberlite XAD-7 (150mg) (Sigma, St Louis, MO) for 30min. After removal of the buffer, the XAD-7 was washed twice with 1ml 1% acetic acid before being slurried two times with 1ml dichloromethane for 30min. The combined dichloromethane fractions were reduced to dryness with nitrogen gas. Then, samples were dissolved in 210 µl methanol and diluted to 700 µl d.i. H2O with 0.1% formic acid. The sample was filtered with a syringe filter (0.2 µm).

The iPA was analyzed using an indirect enzyme-linked immunosorbent assay (ELISA) as described by Zhang and Ervin (2004). Briefly, wells of a 96-unit plate were coated with 100 µl per well of iPA conjugated to bovine serum albumin (BSA) (1:10 000 dilution), incubated overnight at 4ºC, emptied, and washed three times with phosphate buffered saline (PBS, 50mM, pH 7.2)-Tween-20 (PBS containing 0.05% Tween 20). The reaction was ‘blocked’ with 200 µl of 1% BSA in PBS (37ºC, 30min) to prevent nonspecific protein absorption. After the plate was washed twice with PBS-Tween, 50 µl of the iPA extract or iPA standard and 50 µl of the antibody iPA (1:200 dilution) were added to the wells and the plate was incubated at 37ºC for 60min, emptied, and washed three times with PBS-Tween. A series of iPA concentrations (0, 3.13, 6.25, 12.5, 25, and 50ng ml^-1^) were made for a standard curve. To each well, 100 ul of alkaline phosphatase-labeled goat anti-mouse IgG (1:1 000 dilution; Sigma, St Louis, MO) was added and the plate were incubated at 37ºC for 60min. After three washes with PBS Tween, 100 µl of substrate solution (3mg ml^-1^ of p-nitrophenyl phosphate in 10% diethanolamine with 0.5mM MgCl2, pH 9.8) were added to each well and the plate was incubated at 37ºC for 30min. The color reaction in each well was determined by measuring absorbance at 405nm with a microplate reader. Isopentenyl adenosine concentration was calculated on the basis of the standard curve after logarithmic conversion of the data.

### trans-ZR analysis using liquid chromatography-tandem mass spectrometry

The content of trans-zeatin riboside (trans-ZR) was analyzed using liquid chromatography-tandem mass spectrometry (LC-MS/MS) (Alvarez *et al.*, 2008). Root tissues (2.0g) were freeze-dried and ground to powder using mortar and pestle in liquid nitrogen. To each ground sample, 900 µl of ice-cold methanol/acetonitrile (MeOH/ACN, 1:1 [v/v]) and 10 µl of a 2.5 µM deuterium-labeled standard ([d_5_]-trans-zeatin riboside) was added, and the sample was homogenized with the TissueLyser II (QIAGEN, Valencia, CA) for 5min at a frequency of 20 Hz/s, then centrifuged at 16 000 ×*g* for 10min at 4°C. The supernatant was transferred to a new 2ml tube and the pellet was re-extracted as previously described. The second supernatant was combined to the first one and dried down. The dried pellet was dissolved in 200 μl of 30% [v/v] methanol, then centrifuged again to remove un-dissolved material and the supernatant was transferred to vial for LC-MS/MS analysis. The injected volume of the sample was 50 µl.

The LC-MS/MS system used is composed of a Shimadzu LC system with two Shimadzu solvent delivery pumps (LC20AD) and autosampler (SIL20AC) with a 100 ul sample loop (Shimadzu, Kyoto, Japan), and a Valco two-position diverter valve (VICI, Houston, TX). This LC system is interfaced with an AB Sciex 4 000 QTRAP mass spectrometer equipped with a TurboIonSpray (TIS) electrospray ion source (SCIEX, Framingham, MA). Source parameters were set as follows: curtain gas, 20 arbitrary units (a.u.); source gas 1, 50 a.u.; source gas 2, 50 a.u.; collision activated dissociation, high; interface heater, on; temperature, 550°C; ionspray voltage, +5500. Both quadruples (Q1 and Q3) were set to unit resolution. Analyst software (version 1.5) was used to control sample acquisition and data analysis. The 4 000 QTRAP mass spectrometer was tuned and calibrated according to the manufacturer’s recommendations. The hormone contents were detected using MRM transitions that were previously optimized using a standard and a deuterium-labeled standard. All data were presented as the mean (the average content in g DW) ±SE (the standard error) of four biological replicates.

### Root growth analysis

Following 21 d of drought treatment, roots were washed free of fritted clay to measure total root length and root-to-shoot ratio. For root length measurement, roots were stained with 1% crystal violet solution and scanned with a digital scanner (Epson Expression 1680, US Epson, Inc., Long Beach, CA) to generate high-definition digital images. Images were analyzed using WinRHIZO Basic V.2002 software (Regent Instruments Inc., Quebec, QC, Canada) for root length. To determine root-to-shoot (R/S) biomass ratio, shoots were severed from roots, both tissue types were dried in an oven at 80°C for 7 d, weight determined on a mass balance, and ratio of roots to shoots was calculated. All data were presented as the mean (the average content in g dry weight (DW) or ratio) ±SE of four biological replicates.

### Root physiological analysis

Following 21 d of drought treatment, roots free of fritted clay were collected to quantify root electrolyte leakage (EL) and malondialdehyde (MDA) content. EL was measured according to the procedure described by Blum and Ebercon (1981) and used to indicate cellular membrane stability or membrane status following treatment (Merewitz *et al.*, 2011b; Burgess and Huang, 2014). Roots were rinsed with deionized water to remove exogenous solutes and placed in a test tube containing 30ml deionized water. Tubes were agitated in a conical flask shaker for 12h and the initial conductance (C_i_) of incubation solution measured using a conductivity meter (YSI Model 32, Yellow Springs, OH). Tubes containing root tissue were then autoclaved at 121°C for 20min and again agitated for 12h. The maximal conductance (C_max_) of incubation solution was then measured and EL (%) was calculated as [(C_i_/C_max_)×100].

MDA is the final product of lipid peroxidation in plant tissue and was quantified according to the procedure described by Zhang and Kirkham (1996) with slight modifications. Roots (0.5g) were homogenized in 6ml 0.1% trichloroacetic acid (TCA) and the homogenate was centrifuged at 10 000 ×*g* for 10min. 1ml supernatant was added to 4ml 10% TCA containing 0.5% thiobarbituric acid. The mixture was incubated at 95°C for 30min, quickly cooled on ice, and centrifuged at 10 000 ×*g* for 10min at 4°C. The absorbance of supernatant was measured at 532 and 600nm using a spectrophotometer (Spectronic Instruments, Rochester, NY). The concentration of MDA was calculated using an extinction coefficient of 155mM^−1^ cm^−1^ (Heath and Packer, 1968). All data were presented as the mean (the average content in g DW) ±SE of four biological replicates.

### Histochemical staining for hydrogen peroxide and superoxide in roots

Histochemical staining for the presence of hydrogen peroxide and superoxide was performed following 21 d stress treatment according to procedures described in Thordal-Christensen *et al.* (1997) and Dunand *et al.* (2007), respectively, each with slight modifications. To evaluate the presence of H_2_O_2_, roots were stained with 1% (w/v) 3-diaminobenzinidine (DAB; pH 3.8) for 2h and subsequently rinsed with deionized water. To evaluate the presence of O_2_^-^, roots were stained with 2mM nitroblue tetrozolium (NBT) in 20mM phosphate-buffered saline (PBS; pH 6.8) for 30min and subsequently rinsed with deionized water. DAB- or NBT-stained roots were visually observed using an Olympus FSX100 Bio-imaging navigator (Central Valley, PA) and pictures were captured using bright-field single-shot mode at ×4.2 magnification.

### Quantification of reactive oxygen species in roots

The production rate of O_2_^-^ was measured according to the procedure described by Bian and Jiang (2009) with slight modifications. Root tissues (0.5g) were ground to powder in liquid nitrogen, homogenized in 1ml 50mM Tris-HCl (pH 7.5), and centrifuged at 5 000g for 10min at 4°C. 200 μl supernatant was added to 800 μl 0.5mM 3-bis(2-methoxy-4-nitro-5-sulfophenyl)-2H-tetrozolium-5-carboxanilide inner salt (XTT). XTT reduction was recorded once per minute for 3min at 470nm using a spectrophotometer (Spectronic Instruments, Rochester, NY) and the background absorbance was corrected with 50 units of superoxide dismutase (SOD). The O_2_^-^ production rate was calculated using a 2.16×10^4^ M^−1^ cm^−1^ extinction coefficient and expressed as µmol O_2_^-^ min^−1^ g^−1^ DW (Sutherland and Learmonth, 1997).

The content of H_2_O_2_ content was measured according to the procedure described by Zhou *et al.* (2006) with slight modifications. Ground root tissues (0.5g) were homogenized in 5ml 5% (w/v) TCA and the homogenate was centrifuged at 10 000 ×*g* for 20min at 4°C. The supernatant was adjusted to pH 8.4 with 17M ammonia solution, briefly centrifuged to remove large particles, and divided into 1ml aliquots. To a single aliquot, 8 μg catalase was added, to serve as the blank. To each aliquot was added 1ml colorimetric reagent solution containing 10mg 4-aminoantipyrine, 10mg phenol and 5mg peroxidase in 100mM acetic acid buffer (pH 5.6), and the color reaction was incubated for 10min at 30°C. Following incubation, the absorbance was measured at 505nm using a spectrophotometer (Spectronic Instruments, Rochester, NY) and H_2_O_2_ content determined based on standard curve generated with known H_2_O_2_ concentrations. All data were presented as the mean (the average content in g DW) ±SE of four biological replicates.

### Quantification of non-enzymatic antioxidant content in roots

Glutathione (GSH) content was quantified according to the procedure described in Guri (1983) with slight modifications. Frozen root powder (0.5g) was ground to a powder in liquid nitrogen, homogenized with 5ml of 5% ice-cold TCA, and centrifuged at 16 000 ×*g* for 20min at 4°C. The homogenate was titrated to a pH range of 6–8 with 1.5ml 0.1M NaOH. 2.0ml titrated homogenate was added to 0.5ml of 0.2M PBS (pH 7.0), 0.4ml of distilled water, and 0.1ml of dithiobis-2-nitrobenzoic acid (DTNB) and absorbance measured at 412nm using a spectrophotometer (Spectronic Instruments, Rochester, NY). Titrated homogenate containing sodium phosphate buffer, deionized water, but lacking DTNB served as the blank. GSH content was determined based on standard curves generated with known concentrations of GSH.

Free and total ascorbate (ASA) content were quantified according to the procedure described in Ma *et al.* (2008) with slight modifications. Frozen root powder (0.5g) was ground to a powder in liquid nitrogen, homogenized in 8ml 5% (w/v) TCA on ice, centrifuged at 10 000 ×*g* for 10min at 4°C, and the resulting supernatant was used immediately for analysis. For total ASA quantification, the supernatant was incubated in 200mM sodium phosphate buffer (pH 7.4) and 1.5mM dithiothreitol (DTT) for 50min to reduce all dehydroascorbic acid to ASA. Following incubation, 200 μl 0.5% (w/v) N-ethylmaleimide (NEM) was added to remove excess DTT. The resulting solution (0.8ml) was then added to a reaction mixture containing 1ml 10% (w/v) TCA, 800 μl 42% (w/v) o-phosphoric acid, 800 μl 65mM 2,2’-dipyridyl in 70% (v/v) ethanol, and 400 μl 3% (w/v) iron (III) chloride. The reaction was incubated at 42°C for 1h, and absorbance measured at 525nm using a spectrophotometer (Spectronic Instruments, Rochester, NY). Free ASA was measured using the procedure described above with DTT and NEM substituted with 400 μl deionized water. Free and total ASA content were determined based on standard curves generated with known ASA concentrations. All data were presented as the mean (the average content in g DW) ±SE of four biological replicates.

### Quantification of enzymatic antioxidant activity in roots

Enzyme activity of CAT, POD, SOD, APX, DR, MR, and GR was measured according to the procedures described by Zhang and Kirkham (1996). For each CAT, POD, and SOD assay, 0.5g ground root tissue was homogenized in 6ml 50mM sodium phosphate buffer (pH 7.0) containing 0.2mM ethylenediaminetetraacetic acid (EDTA) and 1% (w/v) polyvinylpyrrolidone (PVP) on ice and the homogenates were centrifuged at 15 000 ×*g* for 20min at 4°C. Absorbances were measured at 240, 470, and 560nm for CAT, POD, and SOD, respectively, using a spectrophotometer (Spectronic Instruments, Rochester, NY). For each APX, DR, MR, and GR assay, 0.5g ground root tissue was homogenized in 6ml 25mM sodium phosphate buffer (pH 7.8) containing 0.2mM EDTA and 1% (w/v) PVP and the homogenates were centrifuged at 15 000 ×*g* for 20min at 4°C. Absorbances were measured at 290, 265, 340, and 340nm for APX, DR, MR, and GR, respectively, using a spectrophotometer (Spectronic Instruments, Rochester, NY). All data were presented as the mean (the average content in mg protein) ±SE of four biological replicates.

### Gene expression analysis of enzymatic antioxidants in roots

Gene expression analysis was performed by quantitative reverse transcriptase polymerase chain reaction (qRT-PCR). Total RNA was isolated from root tissue using TRIzol reagent (Life Technologies, Grand Island, NY) and treated with DNase (TURBO DNA-free kit; Life Technologies, Grand Island, NY) to remove contaminating genomic DNA. Using a high-capacity cDNA reverse transcription kit (Life Technologies, Grand Island, NY), 2 μg total RNA was reverse-transcribed and the synthesized cDNA was amplified in a StepOnePlus Real-Time PCR system (Life Technologies, Grand Island, NY) using the following parameters: pre-heat cycle of 95°C for 3min, 40 cycles of 95°C denaturation for 30s, and 60°C annealing/extension for 60s. Power SYBR Green PCR Master Mix (Life Technologies, Grand Island, NY) was the intercalating dye used to detect gene expression level. Gene name, accession number, forward and reverse primer sequences are provided in Table 1. A melting curve analysis was performed for each primer pair to confirm its specificity. *Actin* was used as the reference gene, since its expression was constant throughout treatments. A ΔΔCt method was used to calculate the relative expression level between genes of interest and reference gene, respectively. All transcript levels were expressed as the mean (the average relative expression level) ±SE of four biological replicates.

**Table 1.** Primer sequences of genes used in qRT-PCR Proposed gene names, GenBank accession numbers, best BLAST hit names, E-values, and cellular localizations are also listed.

**Gene**	**Accession number**	**Best BLAST hit**	**E-value**	**Localization**	**Primer sequence**	
CuZn-SOD	DV867103	JQ269675.1 (*Triticum aestivum*)	3e-161	Cytosolic	Forward	CACTGGACCTCACTTCAAC
					Reverse	GTAGCAACACCATCCACTC
POD2	DV867327	XM_010230345.1 (*Brachypodium distachyon*)	6e-153	Cytosolic	Forward	CTTCGACAACGCCTACTAC
					Reverse	TTTGCCCATGTTCACCA
CAT1	DY543619	AJ634002.1 (*Schedonorus arundinaceus*)	0	Chloroplast	Forward	TTGCCAATAAGAGGGAGAATG
					Reverse	CGAAGCCGAGCATGTAAG
APX2	GR281667	KP852178.1 (*Beckmannia syzigachne*)	0	Cytosolic	Forward	AGGACATTGTTGCCCTTTC
					Reverse	GCTCCGTGAAGTAAGAGTTG
GR	AB277097	AB277097 (*Hordeum vulgare*)	0	Cytosolic	Forward	GATGGAGGCTACTTGCTTTG
					Reverse	GCTAAGACCCACGACAGATA
MR	DV865077	KC884831.1 (*Triticum aestivum*)	5e-160	Cytosolic	Forward	CCATGAAGCTCTACAACGAG
					Reverse	GTAGAAGTAGGGCAGGTAGT
DR	DV853556	HM125046.1 (*Puccinellia tenuiflora*)	0	Cytosolic	Forward	GAAAGGTGCCTGTGTTTAATG
					Reverse	GTGATGGAGTTGGGTACTTC
ACT2	DY543529	XM_003578821.2 (*Brachypodium distachyon*)	0	Cytosolic	Forward	CCTTTTCCAGCCATCTTTCA
					Reverse	GAGGTCCTTCCTGATATCCA

### Analysis of root respiration rate

Root respiration rate was measured according to the procedure described by Rachmilevitch *et al.* (2006) with slight modifications. A subset of roots was detached from shoots, washed free of fritted clay, and immediately transferred into 500ml Buchner flasks containing 400ml half-strength Hoagland’s nutrient solution (Hoagland and Arnon, 1950). To mimic drought stress conditions, polyethylene glycol (PEG) 8 000 was added to nutrient solution to adjust the osmotic potential to −1.0MPa (Lagerwerff *et al.*, 1961; Janes 1974). Subsequently, the nutrient solution with or without PEG 8 000 contained either 200 μM sodium nitroprusside (SNP) to inhibit the cytochrome respiratory pathway or 10mM salicylhydroxamic acid (SHAM) to inhibit the alternative respiratory pathway. Solutions containing SNP or SHAM were maintained as an open-flow system by aerating with circulating pumps (Apollo Enterprises Inc., Oxnard, CA) for 30min, after which time a closed-flow system was created by connecting the terminal air tube to the circulating pump inlet. Vacuum grease and Teflon tape were used to maintain an airtight seal around the rubber stoppers. CO_2_ evolution rate was measured every 30min for 2h by extracting 1ml air samples from the flasks using airtight syringes and resealable septa affixed to flask side arms. Air samples were then injected into a Shimadzu GC-8AIT gas chromatograph (Shimadzu, Kyoto, Japan) equipped with a thermal conductivity detector and a stainless steel column (length: 6 ft; I.D.: 0.08''; O.D.: 1/8'') packed with Porapack Q (80/100 mesh). The temperatures for injector, column, and detector were set at 30°C, 150°C and 150°C, respectively. Helium was used as a carrier gas at a flow rate of 30ml min^−1^. Remaining root tissue was dried in an oven at 80°C for 72h and subsequently weighed on a mass balance. Root respiration rates were expressed as CO_2_ evolution rate in mmol h^−1^ g^−1^ DW.

### Statistical analysis

Significant effects of treatment, plant line and their interactions were determined by two-way analysis of variance (ANOVA) using a statistical program (JMP11, SAS Institute, Cary, NC). The effects of transgene, drought stress and the interaction of the above two factors on the parameters tested were provided as *P*-values in Supplementary Table S1 available at *JXB* online. Differences between treatment mean values were distinguished by Student’s *t*-test at the 0.05 probability level.

## Results

### Differential root cytokinin content, root growth and physiological responses to drought stress

**Fig. 1.**
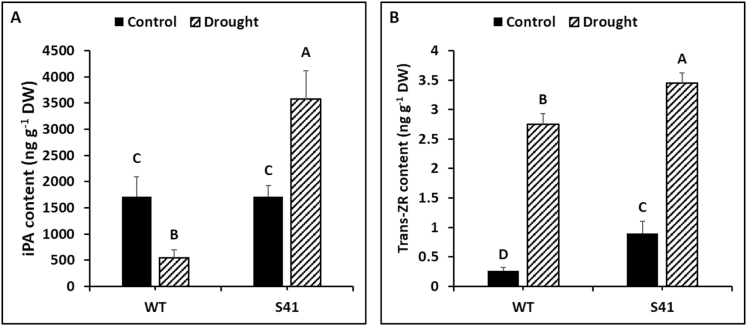
Cytokinin (iPA and trans-ZR) content in roots of WT and transgenic S41 plants under control or drought stress conditions: (A) iPA content, (B) trans-ZR content. Data shown are the mean ±SE of four biological replicates. Letters above bars indicate significant differences at the *P*≤0.05 level.

Cytokinin content varied between the WT and transgenic plants under both well-watered and drought conditions. iPA content was not significantly different between WT and S41 under well-watered conditions (Fig. 1A). Root iPA content decreased under drought stress in WT whereas it increased in S41 and S41 roots had significantly higher amounts of iPA than the WT under drought stress. The trans-ZR content was significantly higher (2.4-fold) in roots of S41 than the WT under well-watered conditions (Fig. 1B). Under drought stress, both WT and S41 had significantly higher ZR contents (2.6 fold and 3.25 fold, respectively), while S41 root also had significantly greater (25%) ZR content than WT roots (Fig. 1B).

**Fig. 2.**
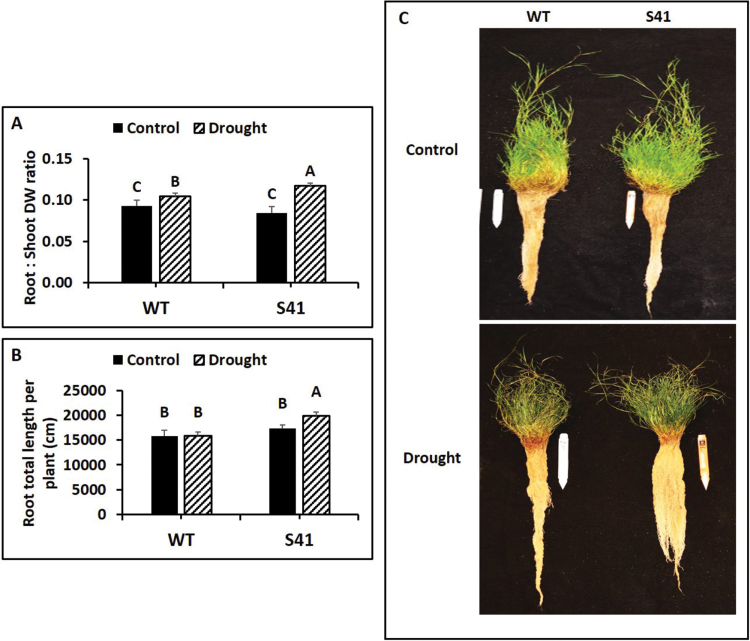
The dry weight (DW) ratio of root to shoot (R/S) (A), total root length per plant (B) and representative plants (C) of WT and S41 under control or drought stress. Data shown are the mean ±SE of four biological replicates. Letters above bars indicate significant differences exist at the *P*≤0.05 level.

The R/S ratio (Fig. 2A) and total length (Fig. 2B) did not differ between the WT and S41 under well-watered conditions. At 21 d of drought stress, root total length and R/S ratio was significantly higher (25% for root length and 12% for R/S) in S41 compared to WT. Under drought stress, WT plants had more elongated root systems, while S41 had more abundant root systems, compared with respective controls. (Fig. 2C).

**Fig. 3.**
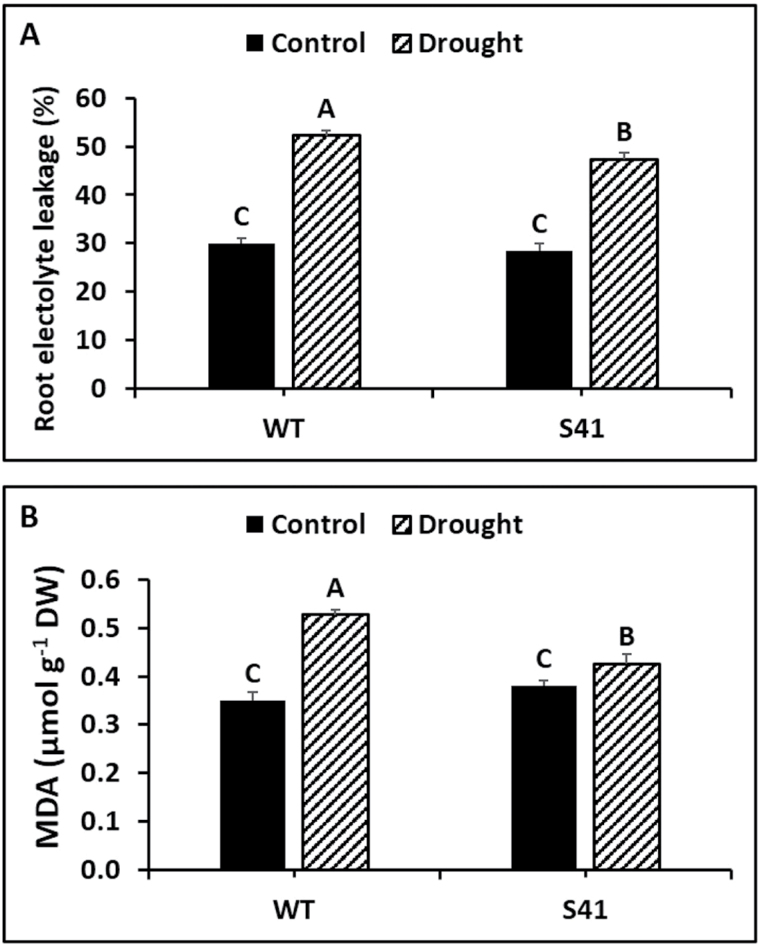
Root electrolyte leakage (A) and MDA content (B) of WT and S41 following control or drought stress. Data shown are the mean ±SE of four biological replicates. Letters above bars indicate significant differences exist at the *P*≤0.05 level.

Root EL did not differ between plant lines under well-watered conditions (Fig. 3A). Root EL increased by 74 and 66% for WT and S41, respectively, at 21 d of drought stress compared to their respective well-watered control. Root EL was significantly lower (9%) in S41 compared to WT under drought stress.

Root MDA content was not significantly different between plant lines under well-watered conditions (Fig. 3B). In response to drought stress, root MDA content increased significantly in both WT (by 50%) and S41 (by 12%) compared to their respective well-watered controls (Fig. 3B). Root MDA content was significantly lower (19%) in S41 compared to WT following drought stress.

### Differential levels of ROS production under drought stress

**Fig. 4.**
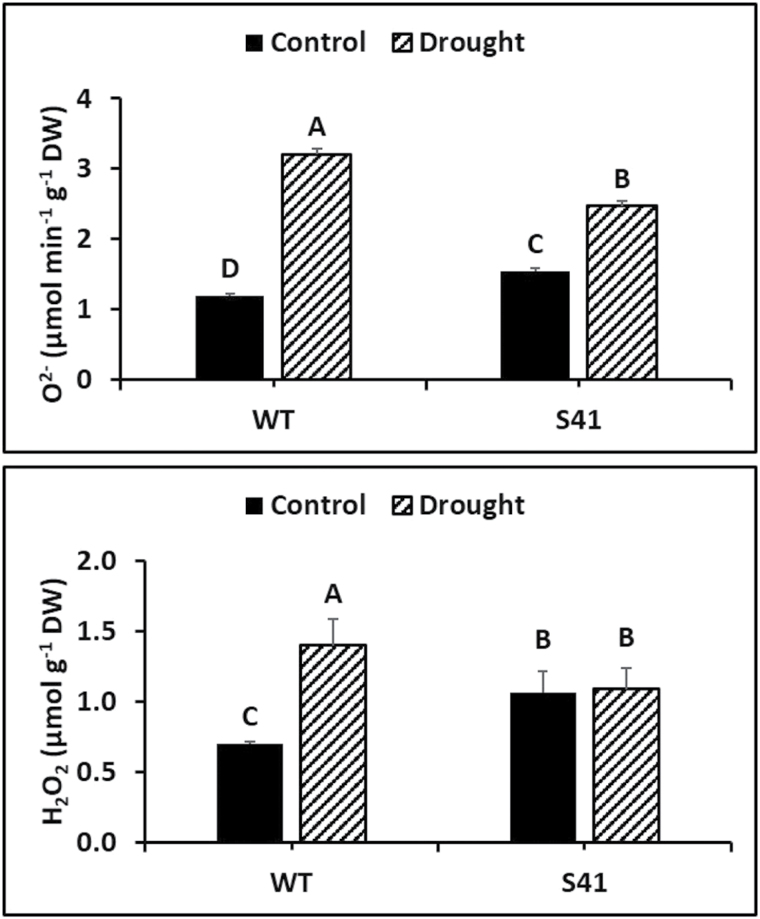
O_2_^-^ production rate (A) and H_2_O_2_ content (B) of WT and S41 following control or drought stress. Data shown are the mean ±SE of four biological replicates. Letters above bars indicate significant differences exist at the *P*≤0.05 level. Bar, 100 μm.

Under well-watered conditions, root O_2_^-^ production rate in S41 was significantly higher than that in WT. Root O_2_^-^ production rate increased 2.7- and 1.6-fold for WT and S41, respectively, at 21 d of drought stress compared to their respective well-watered controls (Fig. 4A). Roots of S41 maintained significantly lower O_2_^-^ production rate (23%) in S41 compared to WT under drought stress.

Under well-watered conditions, root H_2_O_2_ content in S41 was significantly higher than that in WT. Root H_2_O_2_ content increased significantly in WT (by 98%) but did not change significantly under drought stress compared to well-watered conditions (Fig. 4B). Roots of S41 maintained significantly lower (22%) O_2_^-^ production rate in S41 compared to WT under drought stress.

### Non-enzymatic antioxidant content and antioxidant enzyme activities

**Fig. 5.**
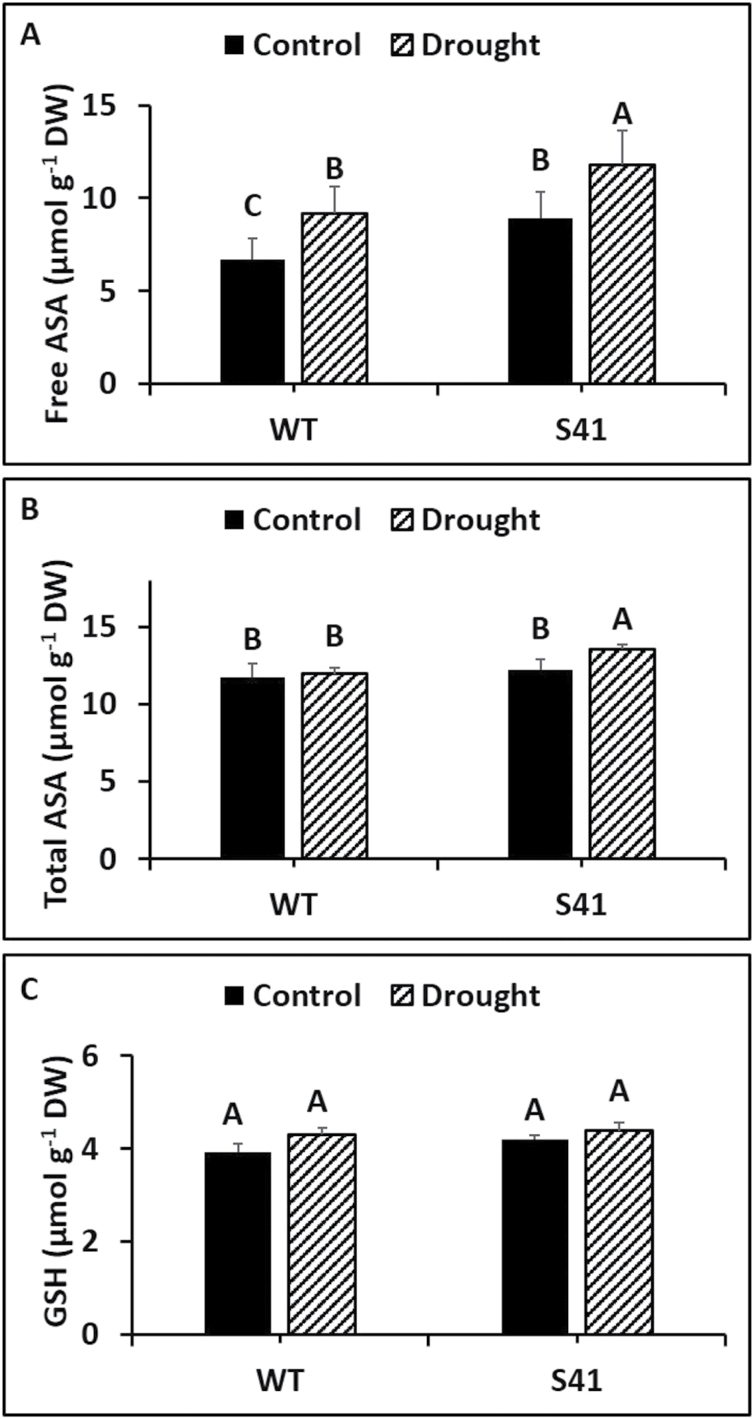
Free ascorbate (A), total ascorbate (B) and glutathione (C) content in WT and S41 roots under control or drought stress. Data shown are the mean ±SE of four biological replicates. Letters above bars indicate significant differences exist at the *P*≤0.05 level.

Root free ASA content was significantly higher (33%) in S41 than WT under well-watered conditions, and remained significantly higher (28%) in S41 under drought stress (Fig. 5A). Root total ASA content did not differ between WT and S41 under well-watered conditions but was significantly higher (14%) in S41 than that in WT under drought stress (Fig. 5B). No significant differences GSH content in roots between WT and S41 under well-watered or drought conditions (Fig. 5C).

**Fig. 6.**
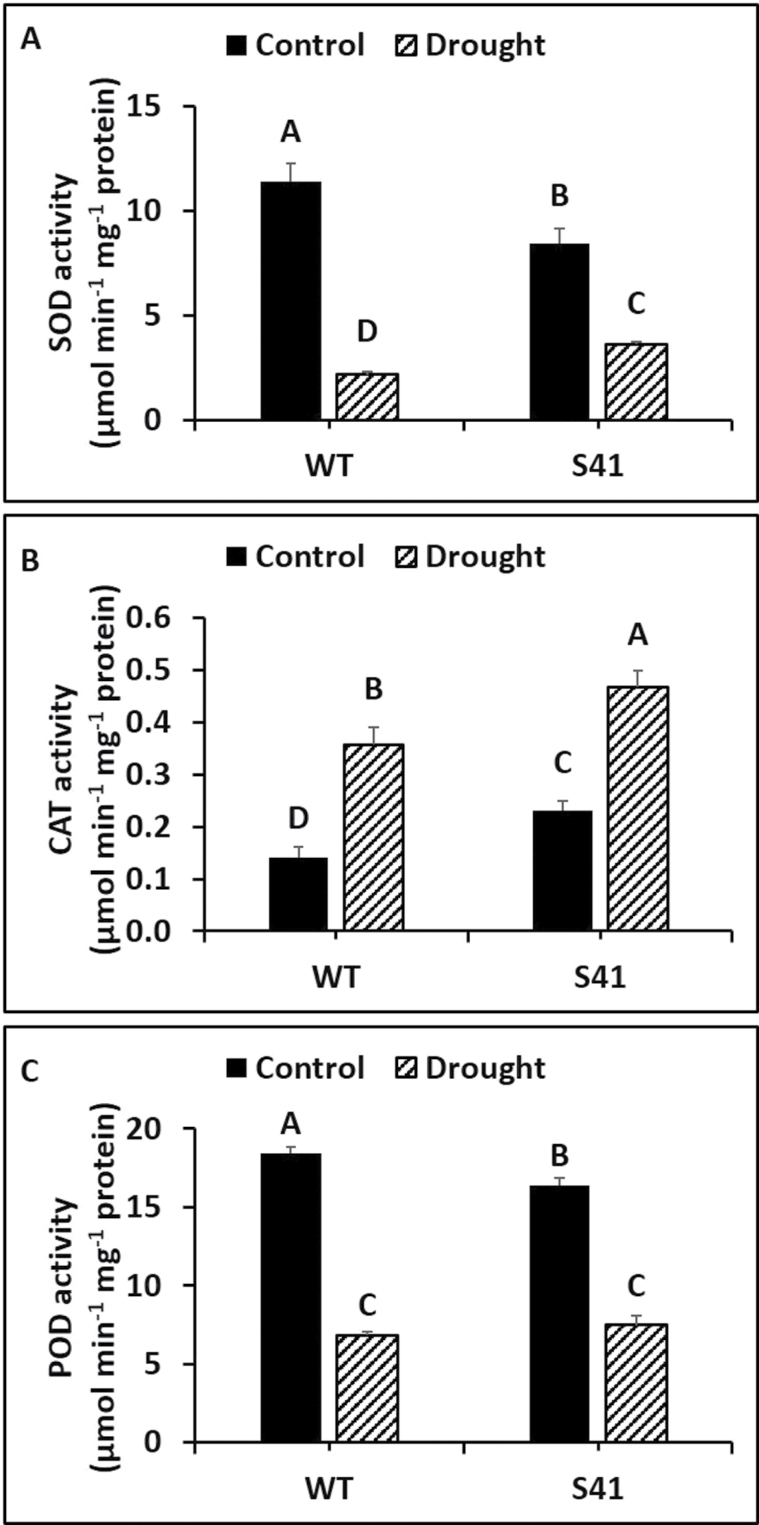
Enzymatic activity of SOD (A), CAT (B) and POD (C) in roots of WT and S41 under control or drought stress. Data shown are the mean ±SE of four biological replicates. Letters above bars indicate significant differences exist at the *P*≤0.05 level.

Root SOD activity was significantly lower in S41 than WT under well-watered conditions, but decreased by 81 and 57% for WT and S41, respectively, under drought stress compared to their respective well-watered controls (Fig. 6A). Roots of S41 exhibited significantly higher (66%) SOD activity than WT roots under drought stress. Root CAT activity was significantly higher in S41 than WT under well-watered conditions, and increased by 2.5- and 2.0-fold for WT and S41, respectively, under drought stress compared to their respective well-watered controls (Fig. 6B). Roots of S41 had significantly higher (31%) CAT activity than WT roots at 21 d of drought stress. Root POD activity was significantly lower in S41 than WT under well-watered conditions, and decreased by 63 and 54%, respectively, due to drought stress treatment (Fig. 6C), while there was no significant difference in POD activity between WT and S41 under drought stress.

**Fig. 7.**
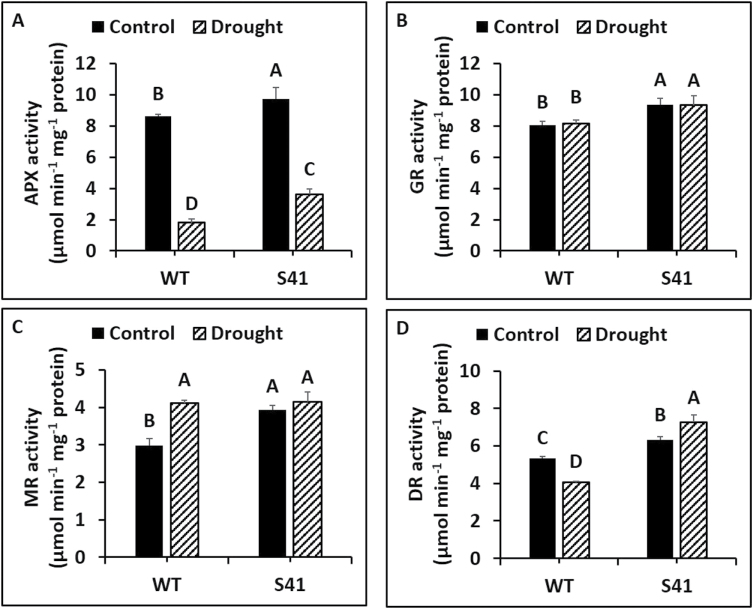
Enzymatic activity of APX (A), GR (B), MR (C) and DR (D) in roots of WT and S41 under control or drought stress. Data shown are the mean ±SE of four biological replicates. Letters above bars indicate significant differences exist at the *P*≤0.05 level.

Under well-watered conditions, root APX, GR, MR and DR activity in S41 were significantly higher than in WT. Root APX activity decreased by 79 and 62% for WT and S41, respectively, due to drought stress (Fig. 7A). Root APX activity was significantly higher (2.0-fold) in S41 compared to WT under drought stress. Root GR activity did not change significantly in response to drought stress for both WT and S41 while it was significantly higher (14%) in S41 than in WT at under both well-watered and drought stress conditions (Fig. 7B). Root MR activity increased by 38% for WT, but did not change in S41 during drought stress and did not differ between WT and S41 at 21 d of drought stress (Fig. 7C). Root DR activity decreased by 24% for WT but increased by 15% for S41 due to drought stress treatment (Fig. 7D). Root DR activity was significantly higher (79%) in S41 compared to WT under drought stress.

### Antioxidant enzyme gene expression

**Fig. 8.**
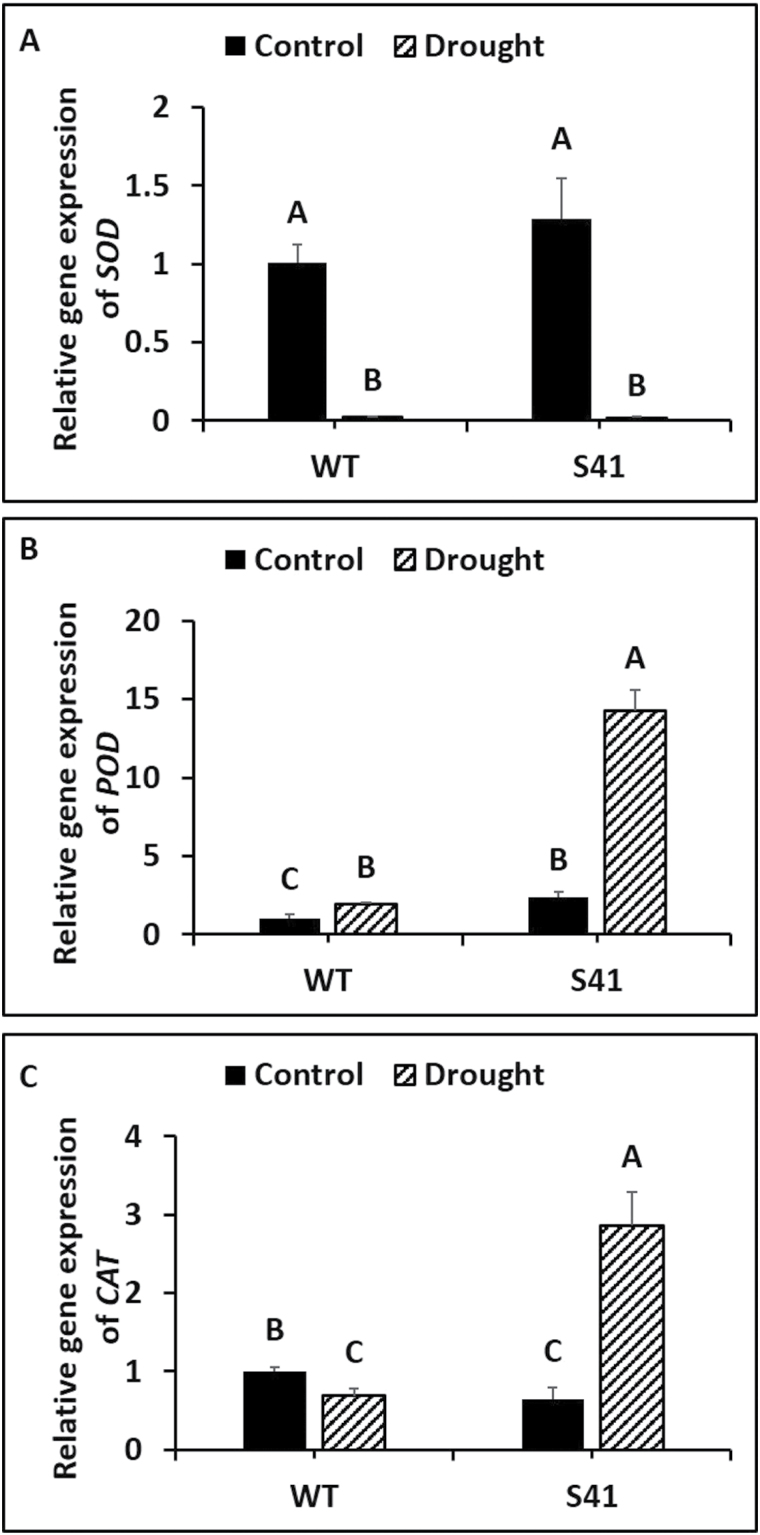
Transcript levels of *SOD* (A), *POD* (B) and *CAT* (C) in roots of control or drought-stressed WT and S41. Data shown are the mean ±SE of four biological replicates. Letters above bars indicate significant differences exist at the *P*≤0.05 level.

Antioxidant enzyme gene transcript levels exhibited significant differences between WT and S41 in response to drought stress treatment. Root *SOD* expression level was significantly down-regulated for WT (by 97%) and S41 (by 98%) due to drought-stress treatment compared to respective well-watered controls (Fig. 8A). Root *SOD* expression level did not differ between WT and S41 under well-watered conditions or drought stress. Root *POD* expression was significantly up-regulated for WT (by 1.9-fold) and S41 (by 6.0-fold) due to drought stress treatment compared to respective well-watered controls and was significantly higher (7.4-fold) in S41 compared to WT under drought stress (Fig. 8B). Root *CAT* expression was significantly down-regulated for WT (by 31%) and up-regulated for S41 (by 4.5-fold) due to drought stress treatment compared to respective well-watered controls (Fig. 8C). Root *CAT* expression was significantly higher (4.1-fold) in S41 compared to WT at 21 d of drought.

**Fig. 9.**
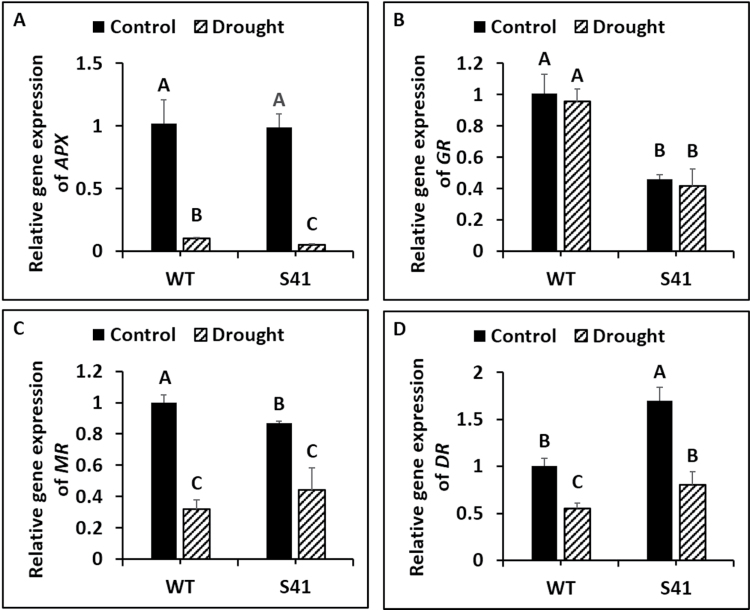
Transcript levels of *APX* (A), *GR* (B), *MR* (C) and *DR* (D) in roots of WT and S41 under control or drought stress. Data shown are the mean ±SE of four biological replicates. Letters above bars indicate significant differences exist at the *P*≤0.05 level.

Root *APX* expression was significantly down-regulated for WT (by 89%) and S41 (by 95%) due to drought stress treatment compared to respective well-watered controls (Fig. 9A). Root *APX* expression was significantly lower (49%) in S41 compared to WT following drought stress. Root *GR* expression did not change in response to drought stress for WT or S41 while it was significantly lower (56%) in S41 compared to WT following drought stress (Fig. 9B). Root *MR* expression decreased by 68 and 54% for WT and S41, respectively, due to drought stress treatment, and did not differ between WT and S41 (Fig. 9C). Root *DR* expression level decreased by 48 and 53% for WT and S41, respectively, due to drought stress treatment. It was significantly higher (1.5-fold) in S41 compared to WT following drought stress (Fig. 9D).

### ROS production in relation to root respiration

**Fig. 10.**
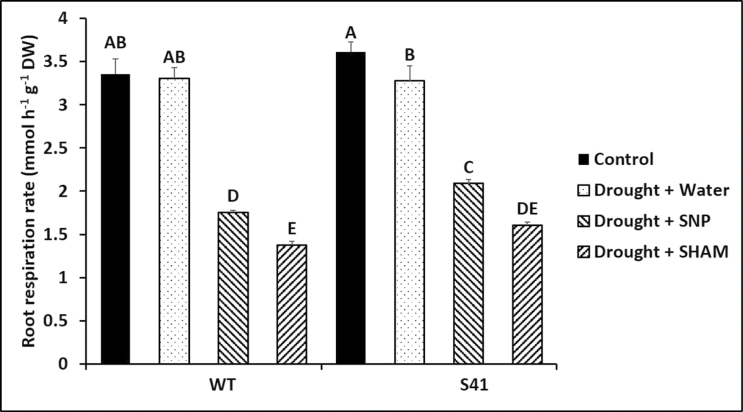
Root respiration rate for WT and S41 under drought stress condition as affected by SNP or SHAM. Data shown are the mean ±SE of four biological replicates. Letters above bars indicate significant differences exist at the *P*≤0.05 level.

The addition of SNP to root incubation solutions reduced respiration rates of drought-stressed roots by 47 and 36% in WT and S41, respectively, compared to non-SNP treatment (Fig. 10). The addition of SHAM to incubation solutions reduced respiration rates of drought-stressed roots by 58 and 51% in WT and S41, respectively, compared to non-SHAM treatment (Fig. 10). Roots of S41 exhibited higher alternative respiration rate (SNP-resistant, SHAM-inhibited) under drought stress, compared to WT roots (Fig. 10).

**Fig. 11.**
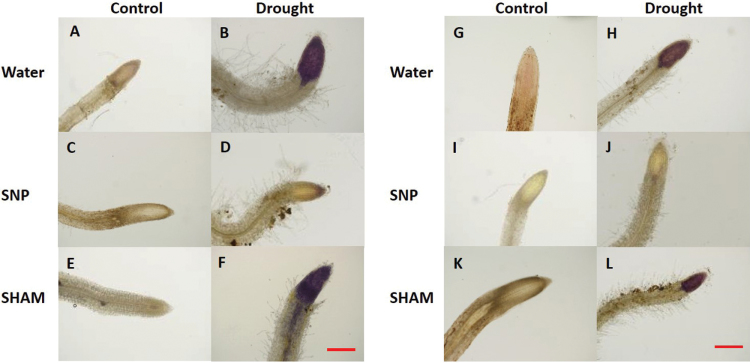
Histochemical staining of water-, SNP- and SHAM-incubated WT (A–F) and S41 (G–L) root tips under control and drought stress conditions using NBT. Bar, 100 μm.

**Fig. 12.**
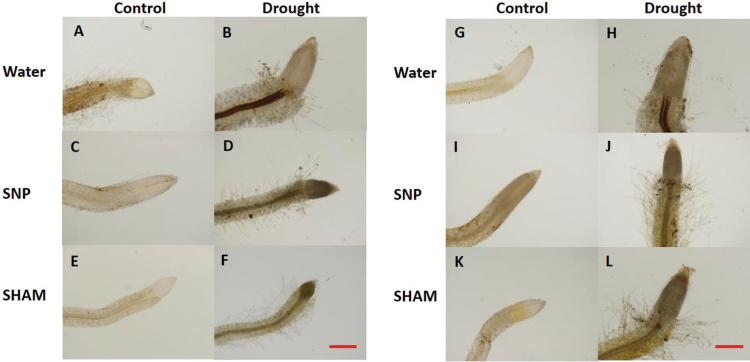
Histochemical staining of water-, SNP- and SHAM-incubated WT (A–F) and S41 (G–L) root tips under control and drought stress conditions using DAB. Bar, 100 μm.

Histochemical staining for O_2_^-^ and H_2_O_2_ in SNP- and SHAM-incubated roots visually depicted the differences in ROS accumulation resulting from changes in respiration rates. Root tips displayed O_2_^-^ accumulation in drought-stressed roots, as shown by increased stain intensity with NBT for O_2_^-^ in WT or S41 roots under drought stress without SNP treatment but stain intensity decreased significantly and was barely visible for root tips treated with SNP treatment; however, SHAM treatment did not appear to be effective to reduce staining for O_2_^-^ in both WT and S41 roots (Fig. 11A–L). DAB staining for H_2_O_2_ (Fig. 12A–L) depict that H_2_O_2_ was accumulated in the vascular tissues of drought-stressed roots of both WT and S41, but the staining intensity decreased in roots of WT and S41 treated with either SNP or SHAM which inhibited cytochrome and alternative respiration, respectively.

## Discussion

The capacity for water uptake by the root system is a key determinant of plant adaptation to drought stress, but severe drought stress typically restricts root production and proliferation, and accelerates root mortality (Sharp *et al.*, 1988; Davies and Zhang, 1991; Davies *et al.*, 2000; Suralta and Yamauchi, 2008). Understanding metabolic factors regulating root growth responses to drought stress is critically important for improving plant drought tolerance. Cytokinins are known to regulate plant responses to drought stress (Hare *et al.*, 1997; Zhang and Ervin, 2004; Rivero *et al.*, 2007, 2009). While multiple types of CK may be present in plants, the predominant types of CK in grasses are isopentenyl adenine (iPA) and zeatin riboside (ZR), which are related to plant stress responses (Xu *et al.*, 2009). In our previous studies, transgenic creeping bentgrass overexpressing *SAG12-ipt* showed strong *ipt* expression in leaves and roots under drought stress, which corresponded to an increase in endogenous iPA and ZR content in leaves and roots (Merewitz, 2010, 2011a). In the present study, increases in both iPA and ZR content were also detected in roots of *SAG12-ipt* transgenic creeping bentgrass. Increasing endogenous CK content has been positively associated with improved plant drought tolerance in bentgrass species based on evaluating shoot growth and physiological activities (DaCosta and Huang, 2007; Merewitz *et al.*, 2010).

In this study, *SAG12-ipt* transgenic creeping bentgrass not only had higher total root length but also greater R/S biomass ratio compared to the WT under drought stress, suggesting that increasing CK in the transgenic plants preferentially promoted root growth over shoot growth when plants were exposed to drought stress and may be associated with CK-regulation of biomass partitioning between shoots and roots. In contrast, the vast majority of research suggests increasing CK content negatively affects root growth of dicot species, such as for Arabidopsis and tobacco (*Nicotiana tabacum*) (Li *et al.* 2006; Laplaze *et al.* 2007; Kuderová *et al.* 2008). Morphology of the fibrous root system in monocots (such as grass species) differs from that of tap root systems of dicots (such as Arabidopsis) which may exhibit unique responses to CK. Several other studies in grass species also reported positive effects of CKs on root growth (Merewitz *et al.*, 2010; Zalewski *et al.*, 2010). For example, RNAi silencing of *HvCKX1* encoding cytokinin oxidase controlling CK degradation in barley (*Hordeum vulgare*) increased root weight and root length and the lower CKX activity or higher CK content is correlated with greater root mass (Zalewski *et al.*, 2010). The regulatory roles of CK for controlling root growth in grass species may differ from the dicot tap root system but underlying mechanisms of CK promoting grass root growth, particularly in response to drought stress, deserves further investigation

Our previous studies found that roots of *SAG12-ipt* plants with increasing CK content maintained significantly higher root viability and metabolic activities as measured by the triphenyl tetrazolium chloride reduction method during prolonged drought stress (Merewitz *et al.*, 2011a). In the present study, enhanced cellular membrane stability (lower EL) and decreased membrane lipid peroxidation (lower MDA content) were observed during drought stress, confirming that increasing endogenous CK content through overexpression of *SAG12-ipt* mitigated cellular damages for creeping bentgrass by maintaining membrane integrity. Although transgenic S41 roots had significantly higher H_2_O_2_ and O_2_^-^ production than WT roots under well-watered conditons, the opposite occurred under drought stress (Fig. 4). This suggests that increasing CK content in the transgenic plants may have suppressed ROS accumulation and mitigated cellular membrane damages (lower EL and MDA content) in roots exposed to drought stress. Most previous studies reported CK involvement in stress-induced oxidative defense in leaves, regulating leaf senescence and shoot growth (Chaloupková and Smart 1994; Kurepa *et al.* 1997; Petit-Paly *et al.* 1999; Zhang and Ervin, 2004; Synkova *et al.*, 2006; Rivero *et al.*, 2007, 2009; Zavaleta-Mancera *et al.*, 2007; Merewitz et al., 2010, 2011a). Our study is one of the first to demonstrate increasing CK production could protect roots from drought-induced oxidative damage and maintain better root growth by suppressing ROS levels in root systems due to CK-activation of antioxidant defense mechanisms.

Antioxidant defense encompasses various antioxidant compounds (i.e. ascorbate and glutathione) and enzymes for dismutation of O_2_^-^ (i.e. SOD) and reduction of H_2_O_2_ by CAT, the ascorbate-glutathione cycle (i.e. APX, MR, DR), or peroxidase cycle (POD) (Bowler *et al.*, 1992; Asada 1992; Foyer and Noctor, 2003; Mittler, 2002). Proteomic analysis of leaves and roots for *SAG12-ipt* transgenic creeping bentgrass revealed a maintenance or increased abundance of several ROS-related enzymes, including CAT-1, APX4, and glutathione S-transferase, under drought stress (Merewitz *et al.*, 2011b). The *ipt*-transgenic tobacco plants exhibited differential elevations of antioxidant transcripts and enzyme activity in different plant organs (Rivero *et al.*, 2007). In the current study, the activities of SOD, CAT, APX and DR and transcript levels of CAT, POD, and DR were all significantly greater in S41 roots compared to WT roots exposed to drought stress, suggesting that the antioxidant effects of CK may be due to activating SOD to transform O_2_^-^ to H_2_O_2_ and then converting H_2_O_2_ to H_2_O through CAT and ascorbate-glutathione cycle enzymes (APX and DR). In addition, free and total ASA content were also significantly higher in S41 roots exposed to drought stress, further confirming the activation of ascorbate-glutathione cycle for H_2_O_2_ scavenging. Moreover, CK promoted CAT activity, but decreased APX activity in *Morinda citrifolia* roots (Baque *et al.*, 2010). The greater antioxidant enzyme activities and free and total ASA content along with up-regulated transcript levels of antioxidant enzymes in *SAG12-ipt* transgenic creeping bentgrass suggests that superior antioxidant capabilities involving both enzymatic and non-enzymatic pathways activated by CK could facilitate efficient ROS scavenging and reducing ROS accumulation, thereby mitigating cellular damage to maintain active root growth under drought stress.

Coinciding with essential energy production and carbon metabolism functions, plant respiration also serves as a major source of ROS production in root tissues (Juszczuk *et al.*, 2001; Gill and Tuteja, 2010). In addition to enzymatic and non-enzymatic pathways mentioned above, alternative respiration regulated by alternative oxidase (AOX) also prevents over-production of ROS during cytochrome respiration caused by excessive NADH supply from cell metabolism (Purvis, 1997, Ribas-Carbo *et al.* 2005). Plant species which exhibit superior drought tolerance may acclimate to unfavorable environments by maintaining lower cytochrome respiration rates and higher alternative respiration rate, thereby avoiding excessive ROS production (Burton *et al.*, 1998; Huang and Fu, 2000; Liu and Li, 2005). Nitric oxide (NO) induces AOX activity and alternative respiration by inhibiting cytochrome oxidase activity, which itself is involved in ROS-facilitated stress responses (Millar and Day, 1996). AOX can be an antioxidant enzyme involved in ROS scavenging (Purvis, 1997). In the current study, S41 roots maintained lower cytochrome respiration rates estimated through SHAM-inhibition of alternative respiration but higher alternative respiration rate induced by the treatment of SNP (NO donor) under drought stress. Roots with the SNP treatment also accumulated less O_2_^-^ in root tips due to the suppression of cytochrome respiration and activation of alternative respiration and either SNP or SHAM treatments also produced less H_2_O_2_ under drought stress. Thus, maintaining greater alternative respiration could suppress ROS production in roots of *ipt*-transgenic creeping bentgrass exposed to drought stress, as shown by lower staining intensity of ROS in roots by DAB or NBT. Rivero *et al.* (2009) reported overexpressing *ipt* in tobacco increased the level of transcripts coding for enzymes in the photorespiration pathway in leaves, which subsequently resulted in increased metabolites generated by photorespiration for antioxidant functions. Several studies investigated the exogenous application of synthetic CKs and the inhibition of both cytochrome and alternative respiration in plant species (Musgrave and Siedow, 1985; Musgrave *et al.*, 1987; Vankova *et al.*, 1991). More recently, an activation of AOX1 in the presence of exogenous CKs was observed in tobacco cells, but its direct relationship to ROS scavenging mechanisms remain unclear. (Mlejnek, 2013). To our knowledge, there are no previous studies relating CK-regulation of alternative respiration in roots to antioxidant defense mechanisms. Our study demonstrated that CK could activate the alternative respiration pathway of roots under drought stress, which along with enhanced activities of SOD, CAT, APX, and DR could mitigate H_2_O_2_ and O_2_^-^ production and drought-induced oxidative stress in roots.

In summary, overexpressing *SAG12*-*ipt* and the associated increased cytokinin content promoted root growth of creeping bentgrass under drought stress and mitigated drought-induced cellular damages, as manifested by greater root length, root-to-shoot ratio, less membrane lipid peroxidation and greater cellular membrane stability of roots. CK could mitigate drought damages in roots by suppressing ROS accumulation and accelerating ROS detoxification by promoting non-enzymatic antioxidant (increased ascorbate content) production, and activating SOD, CAT and ascorbate-glutathione cycle enzymes (APX and DR), as well as enhancing alternative respiration pathways. Further investigation is needed regarding the specific mechanisms and signaling pathways for CK interaction with antioxidant enzymes and genes.

## Supplementary data

Supplementary data are available at *JXB* online.

Table S1. *P*-values for the effect of S41 transgene, drought treatment and S41 transgene × drought treatment by two-way ANOVA. 

Supplementary Data
