# Supplementary material for: Enhancing cytokinin synthesis by overexpressing ipt alleviated drought inhibition of root growth through activating ROS-scavenging systems in Agrostis stolonifera
Source: J Exp Bot. 2016 Feb 17;67(6):1979–92. doi: 10.1093/jxb/erw019 (PMC4783374; doi:10.1093/jxb/erw019)
Supplement: Supplementary Data [file supp_67_6_1979__index.html]

Enhancing cytokinin synthesis by overexpressing ipt alleviated drought inhibition of root growth through activating ROS-scavenging systems in Agrostis stolonifera — Enhancing cytokinin synthesis by overexpressing ipt alleviated drought inhibition of root growth through activating ROS-scavenging systems in Agrostis stolonifera — Supplementary Data 

# Enhancing cytokinin synthesis by overexpressing *ipt* alleviated drought inhibition of root growth through activating ROS-scavenging systems in *Agrostis stolonifera*

## Supplementary Data

Data files

- supplementary\_table\_S1.pdf - Supplementary Data
