# Supplementary material for: Enhancing cytokinin synthesis by overexpressing ipt alleviated drought inhibition of root growth through activating ROS-scavenging systems in Agrostis stolonifera
Source: J Exp Bot. 2016 Feb 17;67(6):1979–92. doi: 10.1093/jxb/erw019 (PMC4783374; doi:10.1093/jxb/erw019)
Supplement: Supplementary Data [file supp_erw019_supplementary_table_S1.pdf]

Enhancing cytokinin synthesis by overexpressing *ipt* alleviated drought-inhibition of root growth through activating ROS-scavenging systems in *Agrostis stolonifera*

Yi Xu[1]*, Patrick Burgess[1]*, Xunzhong Zhang[2], and Bingru Huang[1]**

Table S1. P-values for the effect of S41 transgene, drought treatment and the interaction of the above two (S41 transgene × drought treatment) by two-way ANOVA.

**Parameter**	**Effect of S41 transgene**	**Effect of drought treatment**	**Effect of S41 transgene ×drought treatment**
iPA content	<0.001	0.023	<0.001
trans-ZR content	<0.001	<0.001	0.555
Root:shoot DW ratio	0.147	<0.001	<0.001
Root total length per plant	<0.001	0.052	0.052
Root EL	<0.001	<0.001	<0.001
MDA content	<0.001	<0.001	<0.001
O_2^- content	<0.001	<0.001	<0.001
H_2O_2 content	0.913	<0.001	<0.001
Free ASA content	<0.001	<0.001	0.6271

Total ASA content	0.034	0.056	0.056
GSH content	0.121	0.033	0.588
SOD activity	0.022	<0.001	<0.001
CAT activity	<0.001	<0.001	0.796
POD activity	<0.001	<0.001	<0.001
APX activity	<0.001	<0.001	0.361
GR activity	<0.001	0.847	0.946
MR activity	<0.001	<0.001	<0.001
DR activity	<0.001	<0.001	<0.001
SOD transcript abundance	0.067	<0.001	0.064
POD transcript abundance	<0.001	<0.001	<0.001
CAT transcript abundance	<0.001	<0.001	<0.001
APX transcript abundance	0.067	<0.001	0.036
GR transcript abundance	<0.001	0.773	1.000
MR transcript abundance	0.187	<0.001	0.004
DR transcript abundance	<0.001	<0.001	0.031
Root total respiration	0.542	0.005	0.017

Root cytochrome respiration	0.006	<0.001	0.335
Root alternative respiration	<0.001	<0.001	0.055
